# The association between dairy cattle ownership and nutritional status of children under five in rural Bangladesh: a cross-sectional study

**DOI:** 10.3389/fnut.2026.1766658

**Published:** 2026-02-24

**Authors:** Fatema Tuj Zohora Hira, Mohammad Jahangir Alam, Ismat Ara Begum, Md. Asif Iqbal, Andrew M. McKenzie

**Affiliations:** 1 Department of Agribusiness and Marketing, Bangladesh Agricultural University, Mymensingh, Bangladesh; 2 Department of Agricultural Economics, Bangladesh Agricultural University, Mymensingh, Bangladesh; 3 Department of Agricultural Economics and Agribusiness, University of Arkansas, Fayetteville, AR, United States

**Keywords:** child nutritional status, dairy cattle ownership, livestock, stunting, underweight, wasting

## Abstract

**Introduction:**

The role of livestock production has been frequently acknowledged over recent decades. However, the connection between livestock ownership and child nutritional status has not been sufficiently examined. Keeping this in mind, the study aims to examine the relationship between dairy cattle ownership and the nutritional status of children under five in rural households.

**Methods:**

Using data from the Bangladesh Integrated Household Survey 2018, descriptive and multiple linear regression model was employed to investigate these relationships.

**Results:**

The study highlights alarming rates of child malnutrition, with approximately 33% of children under five being stunted, 24% underweight, and 10% wasted. Regression analyses identify significant factors influencing child nutritional outcomes, including maternal education, household size, farm size, and maternal dietary diversity. Educated mothers are more likely to adopt better child-feeding practices, which in turn reduce the incidence of stunting and underweight in children. Furthermore, educational programs targeting women’s nutrition knowledge can empower them to make informed decisions about their diets and those of their children. In conclusion, this study underscores the complex interrelationships among livestock ownership, women’s education, household dynamics, and nutritional security.

**Discussion:**

It recommends a multifaceted policy approach that prioritizes women`s educational empowerment, strengthens prenatal care coverage and birth preparedness, and expands access to improved and safely managed sanitation facilities. By addressing these areas, policymakers can foster a more nutritionally secure environment for children in rural Bangladesh.

## 1 Introduction

The livestock sector in Bangladesh has seen substantial growth and is vital for the country’s food security, nutrition, and employment (1). Livestock contributes about 2% to Bangladesh’s GDP (1.85% GDP at constant prices) and over 16% to the agriculture sector in the fiscal year (FY) 2021–22 (1). The sector has been growing steadily due to government support, improved medical care, artificial insemination, automation, and increased investment. In FY 2021–22, the total number of livestock in Bangladesh surpassed 430 million (1). Livestock includes both ruminants (cattle, buffaloes, sheep, and goats) and poultry (ducks, chickens), with approximately 56 million cattle, buffaloes, sheep, and goats, along with 375.7 million poultry (2).

Meat production in Bangladesh stands at 8.710 million metric tons and is currently self-sufficient, and milk production has steadily increased, reaching 14.068 million metric tons, while egg production totals 23.3763 billion eggs (1, 2). The livestock sector empowers women, creates self-employment, and contributes to foreign exchange earnings. Opportunities exist in modernizing practices, enhancing productivity, and promoting sustainable livestock farming.

Child nutrition is fundamental to overall health, survival, and long-term human development. A child’s nutritional status is influenced by a range of demographic, socioeconomic, and maternal factors (3). Notably, maternal BMI is significantly associated with children’s nutritional outcomes, highlighting the intergenerational nature of undernutrition (58, 60). Undernutrition remains a critical public health challenge and it manifests in multiple forms, including micronutrient deficiencies, stunting, underweight, and wasting (4). It is the most prevalent form of malnutrition and is responsible for an estimated 3.1 million child deaths worldwide each year (59). Child malnutrition remains highly prevalent in developing countries, particularly in Bangladesh, where a considerable proportion of children are stunted or underweight (5). Globally, approximately 45% of deaths among children under five years in low- and middle-income countries are attributable to malnutrition, with severe acute malnutrition substantially increasing vulnerability to infectious diseases such as malaria, pneumonia, and diarrhea (6).

Livestock ownership can enhance children’s nutritional status by increasing access to nutrient-rich animal-source foods (ASFs), such as eggs, dairy, and meat, thereby improving dietary diversity and reducing the risk of stunting (7, 8). Households with high tropical livestock unit (TLU) scores, indicating greater livestock ownership, are associated with approximately 10% lower odds of stunting among children under five years of age (7). Evidence further indicates that children aged 6–12 months consume eggs more frequently in households owning 4–11 poultry, while ownership of 2–3 or ≥4 dairy animals increases dairy consumption by approximately 1.9–2.0 times, and ownership of ≥12 meat animals raises meat consumption by about 1.4 times (8, 9). In addition, income generated from livestock sales enable households to purchase a more diverse diet, further supporting nutritional adequacy. Integrated livestock-based interventions such as poultry production combined with nutrition education have been shown to amplify positive nutritional outcomes among children (8, 10, 62). Overall, empirical evidence demonstrates that well-designed livestock interventions can substantially improve children’s nutritional status (11). Promoting the production and consumption of ASFs can enhance dietary diversity and improve household income, thereby increasing access to a more varied diet (11). Households receiving livestock interventions have reported generating more income and creating additional employment opportunities (12). Such interventions could serve as pathways to improving livelihoods and achieving nutritional goals (11, 13).

Limited access to financial resources can constrain women’s ability to provide adequate nutrition for young children or to seek appropriate healthcare services (14, 15). However, livestock farming can enhance women’s empowerment by increasing their access to, control over, and decision-making authority regarding income and market participation (16–18). Women’s empowerment has been identified as a critical determinant of optimal child nutrition, particularly during the first 1,000 days of life (19). Women’s ownership and control of livestock assets represent a distinct and important pathway to improved child nutritional outcomes (20, 21). Greater involvement of women in livestock production has also been associated with improved livestock management practices and enhanced household food security (22). Consequently, empowering women as key decision-makers within livestock value chains and households is essential for poverty reduction and contributes positively to the social, economic, and nutritional well-being of families (23).

However, current research reveals a promising trend in alleviating chronic malnutrition, shown by a reduction in the percentage of stunted children in Bangladesh (24). Research by Sarker et al. (21); Holland and Rammohan (20); Meuma et al. (25); Pasqualino et al. (8); Hassan et al. (9); Bari et al. (10); Mechlowitz et al. (26), and others has demonstrated that livestock interventions increase the availability of ASF and dietary diversity, while women’s empowerment is associated with improved child nutritional outcomes. Furthermore, Jones et al. (27) found that women’s empowerment not only enhances child nutrition but also contributes to the achievement of multiple Sustainable Development Goals (SDGs). However, these studies rarely isolate the direct impact of livestock ownership on child anthropometric indicators, such as height-for-age z-scores, weight-for-age z-scores and weight-for-height z-scores. In light of this gap, the present study aims to examine the association between dairy cattle ownership and the nutritional status of children under five years of age in rural Bangladesh using nationally representative data from the Bangladesh Integrated Household Survey (BIHS). By examining potential pathways linking livestock ownership to child nutrition, this study contributes to policy-relevant discussions on the roles of maternal education, dietary diversity, and sanitation in improving child health outcomes.

## 2 Methods

### 2.1 Data source

Data from the Bangladesh Integrated Household Survey (BIHS) 2018 were used to conduct this study (28). The BIHS is a nationally representative dataset of rural Bangladesh conducted by Data Analysis and Technical Assistance Limited under the supervision of the International Food Policy Research Institute.

A competent and adequate statistical approach was applied to determine the total BIHS sample survey of 6,503 households in 325 primary sampling units (PSUs), which are villages. The sample size was selected in two stages: first, by selecting PSUs, and second, by selecting households from those PSUs. Among the 6,503 households, 5,406 households are “Nationally Representative (representative of Rural Bangladesh)” at the division level, while the remaining households belong to the “Feed the Future (FTE) Zone”. These households from the FTF zone were not considered in this study.

### 2.2 Measurement/estimation of different exploratory variables

Table 1 illustrates the different independent variables employed in the study, which were selected on the basis of previous literature.

**Table 1:** Estimation of different exploratory variables.

Variable	Unit
Years of schooling of women	Years
Household size	Discrete
Farm size	Decimal
Women’s dietary diversity score	Discrete
Have dairy cattle	Dichotomous
Share toilet	Dichotomous
Employment status	Dichotomous
Own mobile phone	Dichotomous
The birth weight of a child	Dichotomous
Nutritional knowledge	Categorical (ordered)

Dairy cattle ownership is defined as whether a household owns at least one dairy cow at the time of the survey. Reproduced from (28), licensed under CC BY 4.0.

A validated questionnaire was used to assess mothers’ nutritional knowledge (15, 29, 30). This instrument consisted of 11 questions, with each question carrying 1 mark for the right answer (for details, see Supplementary file). The total score for each individual was calculated by summing the responses and dividing by 11 (the total number of questions). This score was then transformed into a percentage by multiplying by 100. The scale was further classified into three categories. Poor if the scale lies between 0 to 50%, fair if the scale lies between 51–75% and good if the scale lies above 75%. This method follows the approach used in a previous study by Armar-Klemesu et al. (31).

### 2.3 Child nutritional status

In this study, children’s height and weight measurements, adjusted for age and sex, were converted into Z-scores based on the National Center for Health Statistics (NCHS) standards. According to the World Health Organization (WHO), height-for-age, weight-for-age, and weight-for-height are used to define stunting, underweight, and wasting, respectively. In this study, all three indicators are utilized to describe the level of child malnutrition and the relationship between child and maternal nutritional status. Weight was measured in kilograms (kg), and height was measured in centimeters (cm).

WHO Anthro software was used to calculate the Z-scores, as it is a validated tool that automatically calculates the child’s age based on the interview date, ensuring accurate results. Malnutrition status can be categorized into different types based on various standards (Table 2).

**Table 2:** Measurement cut-off.

Indicators	Measurement cut-off
Underweight	Weight for age < −2 standard deviations (SD) of the WHO child growth standards median
Stunting	Height for Age < −2 SD of the WHO child growth standards median
Wasting	Weight for height < −2 SD of the WHO child growth standards median
Overweight	Weight for height > + 2 SD of the WHO child growth standards median

Reproduced from (6), licensed under CC BY-NC-SA 3.0 IGO.

### 2.4 Analytical technique

Summary statistics, including standard deviation, mean, maximum, and minimum, were calculated for all continuous variables, and collinearity among the variables was checked. To assess the data distribution, graphical methods such as histograms, normal density plots, and kernel density plots were used.

In this study, multiple linear regression models were employed to assess the determinants of child nutritional status. The dependent variables in this study were HAZ, WAZ, and WHZ. Households were classified into three categories: (a) households with children under five, (b) households with livestock ownership and children under five, and (c) households without livestock ownership but with children under five. The multiple regression models allow examination of the relationship between key explanatory variables and child nutritional outcomes while controlling for other factors. Three separate multiple linear regression models were constructed for WAZ, HAZ, and WHZ, and each model was subsequently stratified according to household dairy cattle ownership. This approach ensures that observed associations reflect the effect of variables of interest rather than differences between households.

For the entire household, the empirical equation is:

$$\begin{array}{l} Y_{\left(\text{Childre}n^{\prime }s\;\text{nutritional status}-\mathrm{HAZ}\backslash \mathrm{WAZ}\backslash \mathrm{WHZ}\;\right)}=\\ \beta_{0}+\beta_{1}X_{\left(\text{Have livestock}\right)}+\beta_{2}X_{\left(\text{Year of schooling of women}\right)}+\\ \beta_{3}X_{\left(\text{Household size}\right)}+\beta_{4}X_{\left(\text{Farm size}\right)}+\beta_{5}X_{\left(\text{WDDS}\right)}+\\ \beta_{6}X_{\left(\text{Employment status}\;\right)}+\beta_{7}X_{\left(\text{Share toilet}\right)}+\beta_{8}X_{\left(\mathrm{Own}\;\text{mobile phone}\right)}+\\ \beta_{9}X_{\left(\text{Birth weight of}\;a\;\text{child}\right)}+\beta_{10}X_{\left(\text{Nutritional knowledge}\right)} \end{array}$$

To empirically analyse the differences between households with livestock and without livestock, the following model was specified:

$$\begin{array}{l} Y_{\left(\text{Childre}n^{\prime }s\;\text{nutritional status}-\mathrm{HAZ}\backslash \mathrm{WAZ}\backslash \mathrm{WHZ}\;\right)}=\\ \beta_{0}+\beta_{1}X_{\left(\text{Year of schooling of women}\right)}+\beta_{2}X_{\left(\text{Household size}\right)}+\\ \beta_{3}X_{\left(\text{Farm size}\right)}+\beta_{4}X_{\left(\text{WDDS}\right)}+\beta_{5}X_{\left(\text{Employment status}\;\right)}+\\ \beta_{6}X_{\left(\text{Share toilet}\right)}+\beta_{7}X_{\left(\mathrm{Own}\;\text{mobile phone}\right)}+\\ \beta_{8}X_{\left(\text{Birth weight of}\;a\;\text{child}\right)}+\beta_{9}X_{\left(\text{Nutritional knowledge}\right)} \end{array}$$

## 3 Results

### 3.1 Descriptive statistics of different exploratory variables

#### 3.1.1 Nutritional status of children

Table 3 presents the summary statistics of Z-scores derived from the anthropometric data of the children. The mean HAZ is −1.467, indicating that, on average, linear growth falls below the WHO reference median, reflecting a general growth deficit. The mean WAZ (−1.264) and WHZ (−0.63) indicate an average nutritional deficit relative to the WHO reference median.

**Table 3:** Summary statistics of the *z*-score of children under-five.

Variable	Households have children under five		
	Sample size (n)	Mean± std. dev.	Min, max
HAZ	1917	−1.467 ± 1.253	−10.65, 4.9
WAZ	1920	−1.264 ± 1.061	−6.02, 2.99
WHZ	1914	−0.63 ± 1.167	−8.72, 6.78

**Figure 1:**
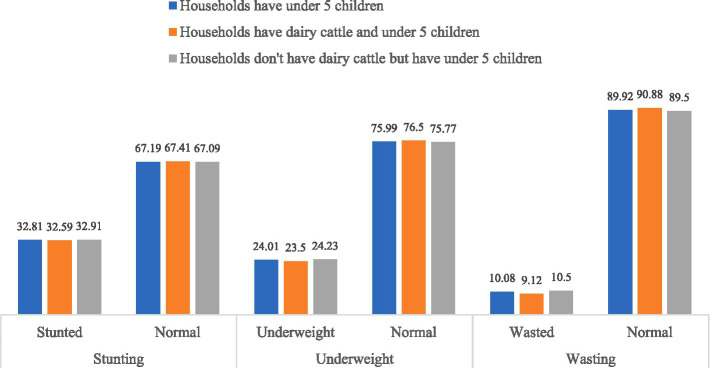
Proportion of children and their nutritional status at different indicators.

Though the mean values of HAZ, WAZ and WHZ provide useful summary information, they do not fully capture the overall nutritional status, as the proportion of children below a cutoff also depends on the distribution of scores. To provide a more detailed view, Figure 1 presents the proportion of children classified according to their nutritional status across the different indicators. After categorizing the Z-scores into three groups -normal, undernourished, and severely undernourished - the study found that approximately 33% of children in households with children under five, those with dairy cattle and children under five, and those without dairy cattle but children under five were stunted. Similar patterns were observed for the other two indicators. Specifically, the prevalence of underweight children was 24%, whereas the prevalence of wasting children was 10%.

#### 3.1.2 Nutritional knowledge of women

To assess maternal nutrition knowledge, the study draws on a set of nutrition-related questions, as shown in Table 4. Overall, maternal nutrition knowledge was relatively high across all household types, with only minor differences between dairy-owning and non-dairy households. Most mothers were aware of recommended practices regarding colostrum feeding, the appropriate timing for introducing liquids and complementary foods, handwashing, encouraging young children to eat, and foods essential for optimal physical growth and cognitive development. However, notable knowledge gaps persist for specific infant feeding practices, particularly those related to early initiation of breastfeeding, breastfeeding frequency, and whether infants under six months should be given water during hot weather. Substantial deficiencies in knowledge were also observed concerning appropriate feeding practices for infants under six months during episodes of diarrhea, whereas the majority of mothers demonstrated adequate knowledge of feeding practices for children older than six months during diarrhea illness.

**Table 4:** Nutrition knowledge-related indicators of women/mothers.

Variables	Households have children under five		Households have dairy cattle and children under five		Households do not have dairy cattle, but have children under five	
	Do not know (%)	Know (%)	Do not know (%)	Know (%)	Do not know (%)	Know (%)
How long after birth should a baby start breastfeeding?	48.26	51.74	51.97	48.03	46.71	53.29
What should a mother do with the “first milk” or colostrum?	5.68	94.32	5.12	94.88	5.92	94.08
How often should a baby breastfeed?	44.66	55.34	44.49	55.51	44.74	55.26
Do you think infants under 6 months of age should be given water if the weather is hot?	47.68	52.32	53.15	46.85	45.39	54.61
When should a baby receive liquids (including water) other than breast milk?	4.99	95.01	5.91	94.09	4.61	95.39
At what age should a baby first start to receive food in addition to breastfeeding?	2.55	97.45	3.15	96.85	2.3	97.7
What should a mother do in regard to child feeding when a child under 6 months has diarrhea?	95.71	4.29	96.85	3.15	95.23	4.77
What should a mother do in regard to child feeding when a child over 6 months has diarrhea?	13.11	86.89	13.78	86.22	12.83	87.17
When should you wash your hands?	0	100	0	100	0	100
What are things you can do to encourage young children to eat their food?	11.14	88.86	12.6	87.4	10.53	89.47
What foods does a young child (<24 months) need to grow and develop their brain?	0.93	99.07	1.18	98.82	0.82	99.18

### 3.2 Determinants of the nutritional status of children

#### 3.2.1 Summary statistics of the predictors of child nutritional status

Table 5 presents descriptive statistics for the key independent variables. Across all households, women’s educational attainment was generally low, with substantial variation in years of schooling. Household size was broadly similar across groups, though slightly larger among dairy-owning households. Farm size also varied considerably, with dairy-owning households typically managing larger farms than non-dairy households. Women’s dietary diversity scores were comparable across household types, indicating similar levels of dietary diversity regardless of livestock ownership. A substantial proportion of households with children under five were engaged in livestock rearing. Regarding sanitation, most households had access to individual toilet facilities, with dairy-owning households less likely to share toilets compared to non-dairy households. The majority of households reported positive empowerment status, particularly among dairy-owning households. Mobile phone ownership among mothers was common across all groups, with slightly higher ownership in non-dairy households. Most children in both household types were born with a birth weight below 2.5 kg. Finally, maternal nutrition knowledge was generally limited, although households with livestock tended to exhibit slightly higher knowledge levels.

**Table 5:** Descriptive statistics of different independent variables.

Variables	Households with children under five			Households with dairy cattle and children under five			Households without dairy cattle but with children under five		
	Mean ± SD	Min, Max	Frequency (%)	Mean ± SD	Min, Max	Frequency (%)	Mean ± SD	Min, Max	Frequency (%)
Years of schooling of women	1.29 ± 3.26	0, 16		1.27 ± 3.12	0, 16		1.30 ± 3.32	0, 16	
Household size	5.13 ± 1.75	2, 17		5.63 ± 1.98	2, 17		4.91 ± 1.59	2, 17	
Farm size (decimal)	8.71 ± 10.63	0.01, 122		10.59 ± 12.09	0.01, 120		7.89 ± 9.82	0.01, 122	
Women’s dietary diversity score	4.06 ± 1.26	1, 9		4.08 ± 1.24	2, 9		4.06 ± 1.27	1, 8	
Have dairy cattle									
No			1,337 (69.64)						
Yes			583 (30.36)						
Share toilet									
No			1,288 (67.08)			425 (72.9)			863 (64.55)
Yes			632 (32.92)			158 (27.1)			474 (35.45)
Employment status									
No			302 (15.73)			28 (4.8)			274 (20.49)
Yes			1,618 (84.27)			555 (95.2)			1,063 (79.51)
Own mobile phone									
No			869 (45.26)			272 (46.66)			558 (41.74)
Yes			1,051 (54.74)			311 (53.34)			779 (58.26)
Birth weight of a child									
Less than 2.5 kg			1,567 (81.61)			485 (83.19)			1,082 (80.93)
More than or equal to 2.5 kg			353 (18.39)			98 (16.81)			255 (19.07)
Nutritional knowledge									
Poor to fair			1,573 (81.93)			493 (84.56)			1,080 (80.78)
Good			347 (18.07)			90 (15.44)			257 (19.22)

#### 3.2.2 Distribution of the outcome variables for the child’s nutritional status

The study conducted the distribution of Z-score for households with children under five, households with dairy cattle and children under five, and households without livestock but with children under five. Results indicate that the distribution for each indicator appears nearly normal, suggesting that multiple linear regression is appropriate for analysing the outcome variables. Based on the results of the normality and heteroscedasticity tests, we conducted model diagnostics and a decomposition analysis to assess the overall fit of the regression model. Multicollinearity among the explanatory variables in the best-fitting model was assessed using the variance inflation factor (VIF). Additionally, the Ramsey RESET test was applied to detect potential model misspecification arising from omitted variables or incorrect functional form.

### 3.3 Determinants of child nutritional status

#### 3.3.1 Determinants of Z-score of height for age (HAZ)

Table 6 presents the regression results for HAZ. Maternal years of schooling are positively and significantly associated with HAZ in both the overall sample households and in households with dairy cattle. Household size shows a negative but significant association with HAZ only among households without dairy cattle. Farm size is positively and consistently associated with HAZ across the overall sample and in dairy-owning households. In contrast, having a shared toilet is negatively associated with HAZ and is significant only in non-dairy households. A one-unit increase in women’s dietary diversity score is significantly associated with higher HAZ across all household categories, holding other factors constant. Furthermore, children with a birth weight of 2.5 kg or higher show a significant positive association with HAZ across all household contexts.

**Table 6:** Determinants of HAZ (multiple linear regression model).

Variables	Households with children under five		Households with dairy cattle and children under five		Households without dairy cattle but with children under five	
	Coef.	St. err.	Coef.	St. err.	Coef.	St. err.
Have livestock (yes)	−0.003	0.064				
Years of schooling of women	0.019**	0.009	0.034*	0.018	0.014	0.011
Household size	−0.027	0.017	0.013	0.029	−0.056***	0.021
Log of farm size	0.032**	0.016	0.025	0.029	0.035*	0.019
Share toilet (Yes)	−0.076	0.061	0.052	0.113	−0.133*	0.072
Employment status (Yes)	−0.127	0.08	−0.199	0.26	−0.118	0.084
Own mobile phone (Yes)	0.07	0.058	−0.008	0.107	0.107	0.068
WDDS	0.078***	0.022	0.104**	0.041	0.065**	0.026
Birth weight of child (> = 2.5 kg)	0.342***	0.078	0.319**	0.144	0.347***	0.093
Nutritional knowledge (good)	0.021	0.081	0.087	0.15	0.008	0.096
Constant	−1.683***	0.148	−1.966***	0.349	−1.492***	0.173
R-squared	0.030		0.038		0.033	
F-test	5.365		2.064		4.877	
Prob > F	0.000		0.031		0.000	

***p < 0.01; **p < 0.05; *p < 0.1.

#### 3.3.2 Determinants of Z-score of weight for age (WAZ)

Table 7 presents the regression results for WAZ. Household size is significant for the overall sample, and for households without dairy cattle, with each additional household member associated with a decrease in children’s WAZ scores. A 1% increase in farm size is associated with an average increase in WAZ in the overall sample. Sharing a toilet is statistically significant but negatively associated with children’s WAZ scores in both the overall sample, and households without dairy cattle. Women’s employment is statistically significant and negatively associated with WAZ in dairy-owning households with children under five. For every one-unit increase in women’s dietary diversity score, children’s WAZ scores increase significantly across all household categories. Children born with a birth weight of 2.5 kg or higher exhibit positive and significantly higher WAZ scores across all household categories. Furthermore, children of women with good nutritional knowledge have significantly higher WAZ scores compared with children of women with poor to fair nutritional knowledge, particularly in overall sample households and non-dairy households.

**Table 7:** Determinants of WAZ (multiple linear regression model).

Variables	Households with children under five		Households with dairy cattle and children under five		Households without dairy cattle but with children under five	
	Coef.	St. err.	Coef.	St. err.	Coef.	St. err.
Have livestock (yes)	0.049	0.053				
Years of schooling of women	0.007	0.008	0.021	0.014	0.001	0.01
Household size	−0.033**	0.014	−0.019	0.023	−0.043**	0.019
Log of farm size	0.023*	0.013	0.016	0.022	0.024	0.017
Share toilet (Yes)	−0.15***	0.052	−0.044	0.097	−0.191***	0.062
Employment status (Yes)	−0.092	0.069	−0.35*	0.206	−0.06	0.073
Own mobile phone (Yes)	0.063	0.048	0.058	0.085	0.061	0.058
WDDS	0.066***	0.019	0.06*	0.035	0.07***	0.022
Birth weight of child (> = 2.5 kg)	0.405***	0.068	0.58***	0.128	0.332***	0.08
Nutritional knowledge (good)	0.153**	0.065	0.116	0.129	0.174**	0.076
Constant	−1.427***	0.124	−1.242***	0.276	−1.388***	0.148
R-squared	0.049		0.077		0.045	
F-test	9.997		4.219		7.587	
Prob > F	0.000		0.031		0.000	

***p < 0.01; **p < 0.05; *p < 0.1.

#### 3.3.3 Determinants of Z-score of weight for height (WHZ)

Table 8 presents the regression results for WHZ. Farm size is significant only in households with children under five, with a 1% increase in farm size associated with a positive change in children’s WHZ score. The presence of a shared toilet is significant and negatively associated with WHZ only in households without dairy cattle. Children born with a birth weight of 2.5 kg or higher show a significant positive association with WHZ across all household categories. Furthermore, children of women with good nutritional knowledge have significantly higher WHZ scores compared with children of women with poor to fair nutritional knowledge, particularly in the overall sample and in non-dairy owning households.

**Table 8:** Determinants of WHZ (multiple linear regression model).

Variables	Households with children under five		Households with dairy cattle and children under five		Households without dairy cattle but with children under five	
	Coef.	St. err.	Coef.	St. err.	Coef.	St. err.
Have livestock (yes)	0.074	0.061				
Years of schooling of women	−0.005	0.009	0.003	0.014	−0.01	0.012
Household size	−0.025	0.015	−0.031	0.025	−0.019	0.02
Log of farm size	0.006**	0.015	0.002	0.028	0.006	0.017
Share toilet (Yes)	−0.134	0.057	−0.055	0.118	−0.158**	0.066
Employment status (Yes)	−0.034	0.079	−0.239	0.223	−0.008	0.085
Own mobile phone (Yes)	0.041	0.054	0.115	0.097	0.002	0.065
WDDS	0.019	0.022	−0.017	0.041	0.035	0.025
Birth weight of child (> = 2.5 kg)	0.31***	0.083	0.536***	0.158	0.217**	0.098
Nutritional knowledge (good)	0.19**	0.074	0.143	0.161	0.212**	0.084
Constant	−0.644***	0.142	−0.285	0.317	−0.709***	0.165
R-squared	0.023		0.046		0.019	
F-test	4.005		2.180		3.444	
Prob > F	0.000		0.022		0.000	

***p < 0.01; **p < 0.05; *p < 0.1.

## 4 Discussion

Our study found that a child’s nutritional status does not appear to be significantly influenced by household ownership of dairy cattle. This contrasts with previous research; for example, Bakhtiar and Hoddinott (32) reported that in rural Bangladesh, dairy cow ownership was associated with an 11% lower likelihood of stunting among young children. Similarly, Muema et al. (25) and Haileselassie et al. (33) found that livestock ownership was linked to a 5–10% reduction in stunting. A possible explanation for the lack of association is that ownership of dairy cattle does not necessarily ensure regular dairy consumption among young children, as households often sell milk to generate income rather than retain it for home consumption.

The household size is negatively associated with the child’s nutritional health. In particular, household size shows a significant negative association with child nutritional outcomes only in households without dairy cattle. In larger families, women’s responsibilities tend to increase, leading to less attention given to children. Additionally, with more family members, there may be less food available for children. Larger families often face more complex challenges related to work and food distribution compared to smaller or nuclear families. However, some studies, such as those found an inverse relationship, which contradicts the findings of this study.

Larger farm size significantly reduces the chances of a child’s malnutrition. A larger farm size provides households with multiple benefits, such as higher income from agricultural produce and greater food availability for household consumption. This, in turn, enables the household to allocate more resources to child health and adopt better feeding practices, leading to improved nutritional outcomes for children under five in rural areas.

The study found that maternal education significantly impacts the likelihood of a child being stunted. In particular, women’s years of schooling are positively and significantly associated with child nutritional outcomes in dairy-owning households. The higher the mother’s education level, the lower the likelihood of child stunting. Educated mothers are generally more aware of their children’s health, which may help reduce stunting (34). Higher education provides opportunities to learn about nutrition and child care through books, classroom activities, other learning resources, and awareness campaigns (such as food or egg days). These resources may encourage mothers to adopt healthier habits and follow recommendations for a diverse diet. Furthermore, an educated mother is more likely to understand the importance of healthy child-feeding practices, such as introducing complementary foods at the appropriate time and exclusively breastfeeding for the first six months of a child’s life, both of which reduce the risk of stunting. Additionally, more educated mothers often belong to higher socioeconomic groups, which allows them to access better healthcare for their children and use household resources more efficiently.

The finding is consistent with Makoka and Masibo (34), who identified 10 years of schooling as a protective threshold in Malawi, Zimbabwe, and Tanzania. A similar result was observed in a study by Chirande et al. (35). In another study, Chowdhury et al., (36) found that approximately one-tenth of children whose mothers had no education were severely stunted. According to Sadika et al. (37), literate mothers are better able to understand and follow health-related messages in print media, as well as medical instructions for treating childhood illnesses and acting on them. This finding aligns with results from numerous other studies (38–45).

Although maternal employment was associated with slightly poorer child nutritional status, this relationship was observed only for WAZ, and the level of statistical significance was weak (*p* = 0.10). No significant associations were found between maternal employment and other anthropometric indicators, including HAZ or WHZ, suggesting that maternal employment does not have a consistent adverse effect on overall child nutrition in this study population. Similar studies by Holland and Rammohan (20) and Ogutu et al. (46) support our findings, highlighting that broader women’s economic empowerment, beyond employment status alone, is linked to a lower prevalence of child malnutrition. However, the findings of Win et al. (47) contradicts our results, indicating a potential adverse effect of maternal employment on child nutrition.

Women’s Dietary Diversity Score (WDDS) is positively associated with child nutritional outcomes in both household strata; although the magnitude of the coefficients is generally larger among dairy-owning households. These findings are consistent with previous studies ((29, 30, 48); and (49)), which highlight that higher WDDS is correlated with improved child growth outcomes, as diverse maternal diets enhance the availability of essential micronutrients for breastfeeding and complementary feeding.

Maternal nutritional knowledge is a significant predictor of a child’s nutritional status. This knowledge helps a mother or woman make informed decisions about what foods are best for her child, how to feed the child, what to do when the child falls ill, and when to introduce diverse foods. Such knowledge is crucial for the growth and development of a healthy child. Several studies have found similar results, supporting the findings of this study (29, 30, 50–53).

Birth weight is an important determinant of a child’s nutritional status. A healthy birth weight makes a child less vulnerable to malnutrition, while low birth weight can increase the risk of various health issues, including long-term stunting. Several studies have shown how low birth weight negatively impacts a child’s nutritional status (54). Our findings are consistent with these previous studies. Furthermore, birth weight exhibits a strong and consistent positive association with child nutritional outcomes across all three groups, indicating that prenatal factors have a robust influence on child nutrition regardless of dairy cattle ownership.

Healthy eating habits alone cannot guarantee better nutritional status in children if sanitation facilities are inadequate. Poor sanitation directly undermines both food intake and health, whereas adequate sanitation is essential for proper nutrition absorption. In this regard, toilet sharing emerged as a critical factor influencing children’s nutritional status. Our study found that stunting was more prevalent among children living in households that shared a toilet, highlighting the potential impact of sanitation on growth outcomes. The negative effect of shared toilet facilities on child nutritional outcomes is consistently stronger and statistically significant among households without dairy cattle. This effect may occur because shared toilets can increase infection risk, compromising gut health, hinder the maintenance of a clean environment, and make children more susceptible to disease transmission. Consistent with this, Chowdhury et al. (36) reported that children from families using unimproved sanitation facilities were more likely to be stunted compared to those from families with flush toilets. Headey and Palloni. (61) demonstrated that improved sanitation could significantly reduce child morbidity and mortality. As part of the WASH framework, poor sanitation is a major driver and indicator of childhood stunting (55, 56). Rahman et al. (57) also found that improved sanitation practices can reduce the risk of childhood stunting compared to households with inadequate sanitation.

## 5 Limitations

The study contributes new insights but also has several limitations. Being cross-sectional, it does not allow for causal inference. Some variables, such as birth weight, rely on maternal recall and may be subject to recall bias, while self-reported information on employment or farm size may introduce social desirability bias. Due to data unavailability, the study could not include the most recent information that primary data collection might have provided. Furthermore, the use of secondary data restricted the inclusion of important determinants of child nutritional status - such as paternal education, household income, WASH conditions, and immunization potentially resulting in residual confounding.

## 6 Conclusion and recommendations

This study examines the nutritional status of children under five in Bangladesh in relation to dairy cattle ownership, highlighting malnutrition as a persistent concern shaped by socioeconomic, maternal, perinatal, and environmental factors. The findings indicate that child undernourishment remains prevalent, with approximately one-third of children under five stunted, one-quarter underweight, and one-tenth wasted. Key determinants, such as maternal education, household size, and farm size, play a significant role in shaping child nutritional outcomes. Educated mothers are more likely to adopt healthier feeding practices, thereby improving child health and reducing stunting. While larger household sizes may present challenges in resource allocation, they can also provide greater support and shared responsibilities, potentially benefiting child nutrition. Conversely, shared sanitation facilities were consistently associated with poorer nutritional outcomes, particularly for WAZ and WHZ, underscoring the influence of environmental health conditions on child growth. Maternal employment exhibited a negative association with child WAZ in households with dairy cattle, suggesting potential trade-offs between women’s labor participation and childcare. Regression results further show that women’s dietary diversity and child birth weight (≥2.5 kg) are strong and robust predictors of all three nutritional outcomes (HAZ, WAZ, and WHZ). Maternal nutrition knowledge positively influenced weight-based indicators (WAZ and WHZ) but showed a limited association with linear growth (HAZ).

Policies and programs that strengthen maternal and newborn health interventions are essential. Improving prenatal care coverage and birth preparedness can reduce the incidence of low birth weight and support healthy child development from early life. Maternal nutrition should be prioritized, as women’s dietary diversity showed a consistently positive and significant association with child nutrition across all household categories. Investments in women’s education should be reinforced, as even modest gains in educational attainment can translate into improved child nutrition outcomes. In addition, expanding access to improved and private sanitation facilities can reduce exposure to infections that undermine child growth. Targeted and integrated interventions addressing maternal health, nutrition, education, and sanitation can foster a more resilient and nutritionally secure population, paving the way for a healthier future for children in Bangladesh.

## 7 Ethical consideration

The study was conducted in accordance with the principles of the Declaration of Helsinki. It utilized publicly available data from the Bangladesh Integrated Household Survey (BIHS), administered by the International Food Policy Research Institute (IFPRI). The BIHS obtained written informed consent from all respondents before data collection. In addition, ethical approval from the Research Ethics Committee of Bangladesh Agricultural University was not required, as the study was based on publicly available secondary data.

## Data Availability

Publicly available datasets were analyzed in this study. This data can be found at: https://bangladesh.ifpri.info/bangladesh-integrated-household-survey.
