# Supplementary material for: The association between dairy cattle ownership and nutritional status of children under five in rural Bangladesh: a cross-sectional study
Source: Front Nutr. 2026 Feb 24;13:1766658. doi: 10.3389/fnut.2026.1766658 (PMC12971449; doi:10.3389/fnut.2026.1766658)
Supplement: Supplementary file 1 [file Data_Sheet_1.pdf]

Appendix

The nutrition knowledge of mothers/ women, as mentioned in the above table, has been derived from several nutrition-related questions. These questions are:

- How long after birth should a baby start breastfeeding?
- What should a mother do with the "first milk" or colostrum?
- How often should a baby breastfeed?
- Do you think infants under 6 months of age should be given water if the weather is hot?
- When should a baby receive liquids (including water) other than breast milk?
- At what age should a baby first start to receive food in addition to breastfeeding?
- What should a mother do in regard to child feeding when a child under 6 months has diarrhea?
- What should a mother do in regard to child feeding when a child over 6 months has diarrhea?
- When should you wash your hands?
- What are things you can do to encourage young children to eat their food?
- What foods does a young child (<24 months) need to grow and develop their brain?

All the above questions/ indicators were binary response types, indicating whether women/mothers had knowledge/awareness or not of each specific question. Positive replies were coded as 1, while negative reactions were coded as 0. This process was conducted following established research methodologies (Hira et al., 2025; Haque et al., 2022 & 2023) to ensure methodological rigor, consistency, and validity of the findings. Various dimensions of breastfeeding practices and maternal caregiving behaviors were assessed using structured questionnaire items and subsequently operationalized as binary variables for analysis. Early initiation of breastfeeding was defined as commencing breastfeeding immediately after birth or within one hour of delivery, coded as 1, with initiation beyond this period coded as 0. Mothers' knowledge of the importance of colostrum was also evaluated, with affirmative responses coded as 1 and negative responses coded as 0. Breastfeeding and maternal caregiving practices were assessed. Breastfeeding on demand

was coded as 1 if mothers reported feeding in response to their baby and 0 otherwise. Maternal opinions on providing water to infants under six months, particularly during hot weather, were recorded, with affirmative responses coded as 1 and non-affirmative responses coded as 0. Awareness of the appropriate timing for introducing liquids or complementary foods was captured, with responses indicating six months or older coded as 1. Maternal caregiving during episodes of infant diarrhea was also evaluated. For children under six months, continuation of breastfeeding was coded as 1, with cessation coded as 0. For children aged six months and older, continued feeding alongside oral rehydration solution (ORS) or home-prepared fluids was coded as 1, with other practices coded as 0. Handwashing practices prior to caregiving were coded as 1 for affirmative responses and 0 for uncertain or negative responses. Maternal strategies to promote healthy eating behaviors in children were recorded, with adoption coded as 1 and non-adoption as 0. Finally, maternal knowledge regarding the nutritional requirements of children under 24 months for optimal physical growth and brain development was assessed and coded accordingly.
